# A Transformer Encoder Approach for Localization Reconstruction During GPS Outages from an IMU and GPS-Based Sensor

**DOI:** 10.3390/s25020522

**Published:** 2025-01-17

**Authors:** Kévin Cédric Guyard, Jonathan Bertolaccini, Stéphane Montavon, Michel Deriaz

**Affiliations:** 1 Information Science Institute, GSEM/CUI, University of Geneva, 1227 Carouge, Switzerland; jonathan.bertolaccini@unige.ch; 2 Veterinary Department of the Swiss Armed Force, 3003 Berne, Switzerland; smontavon@bluewin.ch; 3 Haute Ecole de Gestion Genève, HES-SO, 1227 Carouge, Switzerland; michel.deriaz@hesge.ch

**Keywords:** localization reconstruction during GPS outages, Transformer, bidirectional encoder, deep learning, time series

## Abstract

Accurate localization is crucial for numerous applications. While several methods exist for outdoor localization, typically relying on GPS signals, these approaches become unreliable in environments subject to a weak GPS signal or GPS outage. Many researchers have attempted to address this limitation, primarily focusing on real-time solutions. However, for applications that do not require real-time localization, these methods remain suboptimal. This paper presents a novel Transformer-based bidirectional encoder approach to address, in postprocessing, the localization challenges during GPS weak signal phases or GPS outages. Our method predicts the velocity during periods of weak or lost GPS signals and calculates the position through bidirectional velocity integration. Additionally, it incorporates position interpolation to ensure smooth transitions between active GPS and GPS outage phases. Applied to a dataset tracking horse positions—which features velocities up to 10 times those of pedestrians and higher acceleration—our approach achieved an average trajectory error below 3 m, while maintaining stable relative distance errors regardless of the GPS outage duration.

## 1. Introduction

A widely used approach for 3D localization is the Inertial Measurement Unit (IMU) [1], which typically includes accelerometers, gyroscopes, and occasionally magnetometers. IMUs provide 3D acceleration and orientation data. However, due to inherent noise, IMU-based localization (which requires the double integration of acceleration) is prone to significant temporal drift [2], making reliable positioning feasible for only brief periods. To address this, IMU data are often combined with Global Positioning System (GPS) data through a Kalman Filter (KF) [3].

In outdoor environments, combining IMU and GPS data with a KF enables accurate localization. However, in indoor settings or in environments with poor GPS coverage, this approach is limited by GPS signal issues, such as weak signals or signal loss. Since the sensor’s localization heavily depends on GPS data, its accuracy declines sharply in this type of environment.

Deep-learning-based methods have been developed to provide real-time localization during GPS outages by leveraging information from past data. Although these methods can also offer localization for applications that do not require real-time processing, they have a limitation: they cannot utilize future data. Thus, these approaches lead to suboptimal localization when both past and future data can be used.

In this paper, we present a novel deep learning approach using a Transformer-based bidirectional encoder to reconstruct, in postprocessing, the localization during GPS weak signal phases or GPS outages. This approach employs raw IMU and GPS data, along with the KF output, to predict the velocity. The position is then obtained through the bidirectional integration of these velocity data. We also incorporate position interpolation to ensure smooth transitions between recorded and predicted positions during GPS outages.

The proposed approach is tested using the Alogo Move Pro sensor, a device specifically designed for equestrian applications. This device uses an Inertial Navigation System (INS) that combines IMU and GPS data through a KF, enabling the 3D localization of a horse during training and competition. Based on this localization, the device generates various metrics, such as the stride analysis, power, and maximum jump height. Riders typically review session data after training or competition, allowing for postprocessing and enabling the information from both past and future data to be leveraged when applying localization reconstruction.

The sensor’s outdoor accuracy has been validated [4]. However, in indoor environments, the sensor experiences GPS outages, compromising the precision required for the effective analysis of horse behaviors. GPS outages in the sensor occur in three main cases: at the start, during, or at the end of a session. Additionally, the GPS outage duration can be categorized into short outages (10 to 20 s) and medium outages (30 s to 2 min).

## 2. Related Works

Many researchers have explored ways to reduce noise in IMU data by investigating alternative fusion methods that do not rely on GPS data. Several factors may drive the choice to use only IMU data, including the cost of adding a GPS receiver and the impracticality of the GPS in certain contexts, such as indoor localization within buildings. Sun et al. [5] proposed a two-stage pipeline to address this challenge: first predicting device orientation and then estimating the position based on IMU data from a smartphone. Their approach utilized a Long Short-Term Memory (LSTM) network in the first stage and a bidirectional LSTM network in the second. Chen et al. [6] introduced a method that segments IMU data into windows, applying an LSTM network to learn the relationship between raw acceleration data and the polar delta vector, from which localization is derived. Brossard et al. [7] took a different approach, presenting a Convolutional Neural Network (CNN) to correct gyroscope noise in raw IMU signals.

In another study [8], the authors proposed an extended KF based on a 1D version of ResNet18 to fuse IMU data for localization estimation. A more lightweight adaptation of this approach, compatible with standard smartphones, was developed in [9]. Similarly, Wang et al. [10] proposed a ResNet18-inspired network for fusing IMU data. Cioffi et al. [11], focusing on drones, adopted a Temporal Convolutional Network (TCN), citing studies showing that TCNs perform comparably to Recurrent Neural Networks (RNNs) for temporal sequences, with the added advantages of easier training and deployment on robotic platforms. Rao et al. [12] introduced a contextual Transformer network with a spatial encoder and a temporal decoder to fuse IMU data for velocity prediction, which allowed for localization via velocity integration. Wang et al. [13] achieved high localization accuracy from IMU data using a network with convolution blocks, a bidirectional LSTM, and a Multi-Layer Perceptron (MLP), incorporating an attention mechanism.

Other studies have aimed to improve the fusion of IMU and GPS data, which are traditionally managed through a KF. Hosseinyalamdary [14] presented a deep KF using an LSTM to fuse IMU and GPS data, achieving better localization than the conventional KF. Wu et al. [15] replaced the KF with a TCN for position and velocity prediction, adopting a multitask strategy to reduce prediction errors. Lastly, Kang et al. [16] developed a model combining a CNN with a Gated Recurrent Unit (GRU) to predict the pedestrian velocity.

This literature review indicates that extensive efforts have been made to either predict localization from IMU data alone or enhance GPS and IMU data fusion. However, the problem of GPS signal loss has received limited attention, with most studies addressing GPS outages in real-time applications. As a result, these real-time methods cannot leverage the benefits of a postprocessing bidirectional perspective. While such methods may work for applications not requiring real-time localization, they yield suboptimal results. To address this gap, we propose a deep learning approach for localization reconstruction in postprocessing, utilizing both past and future data to improve the localization accuracy.

## 3. Materials

### 3.1. Data

To develop our deep learning approach, we collected data from the Alogo Move Pro device (from the company Alogo Analysis SA, Le Mont-sur-Lausanne, Switzerland) during outdoor sessions. The data capture a variety of contexts, including training sessions, equestrian trails, and competition courses. In total, 53 sessions were recorded, amounting to 29 h and 4 min of data. The shortest session lasted 27 s, while the longest extended to 49 min and 57 s. The average session duration was 32 min and 54 s, with a standard deviation of 12 min and 48 s. The data capture a variety of contexts: dressage practice (10.4%), flat work practice (31.2%), jumping practice (12.5%), outriding practice (12.5%), racing practice (12.5%), competition (14.6%), and other (6.2%). The sessions were divided into three datasets: training (64%), validation (18%), and testing (18%). The data were split with the intention of preserving the contextual representation in each subset, ensuring that it mirrors the distribution and characteristics of the full dataset.

Each session consists of 100 timestamps per second, and for each timestamp, the device provides the following data:

- A^X^_IMU_, A^Y^_IMU_, A^Z^_IMU_: Acceleration measured by the IMU;
- R^A^_IMU_, R^B^_IMU_, R^C^_IMU_: Rate of rotation measured by the IMU;
- M^X^_IMU_, M^Y^_IMU_, M^Z^_IMU_: Magnetic field measured by the IMU;
- P^X^_GPS_, P^Y^_GPS_, P^Z^_GPS_: Position measured by the GPS;
- V^X^_GPS_, V^Y^_GPS_, V^Z^_GPS_: Velocity measured by the GPS;
- E_GPS_: Position accuracy estimation provided by the GPS (PDOP);
- P^X^_FUSION_, P^Y^_FUSION_, P^Z^_FUSION_: Position computed by the sensor fusion algorithm;
- V^X^_FUSION_, V^Y^_FUSION_, V^Z^_FUSION_: Velocity computed by the sensor fusion algorithm;
- O^A^_FUSION_, O^B^_FUSION_, O^C^_FUSION_: Orientation computed by the sensor fusion algorithm;
- E_FUSION_: Position accuracy estimation provided by the sensor algorithm.

GPS data are refreshed at a frequency of 5 Hz, while IMU and sensor fusion algorithm data are refreshed at 100 Hz. Table A1 presents various statistical details about these features across the entire dataset.

The P_FUSION_, V_FUSION_, and O_FUSION_ values are computed by the sensor fusion algorithm using IMU data (A_IMU_, R_IMU_, and M_IMU_) and GPS data (P_GPS_ and V_GPS_). During GPS outages or periods of weak GPS signals, the accuracy of P_GPS_ and V_GPS_ declines, which affects P_FUSION_, V_FUSION_, and O_FUSION_ due to error propagation. In this study, our primary goal is to accurately reconstruct the P_FUSION_ during these GPS outage phases.

### 3.2. Testing Subdatasets

As outlined in the introduction, there are three primary cases of GPS outages: at the beginning of the session, during the session, and at the end of the session. Additionally, GPS outages can be categorized by duration into two types: short outages (10 to 20 s) and medium outages (30 s to 2 min). To simulate these scenarios, we created three masking schemas to generate testing subdatasets from the original testing dataset:

- **Schema 1**: The beginning of the session is masked.
- **Schema 2**: A section within the session is masked, while the beginning and end of the session remain unmasked (at least 2 s are left unmasked at both the start and end).
- **Schema 3**: The end of the session is masked.

**Figure 1:**
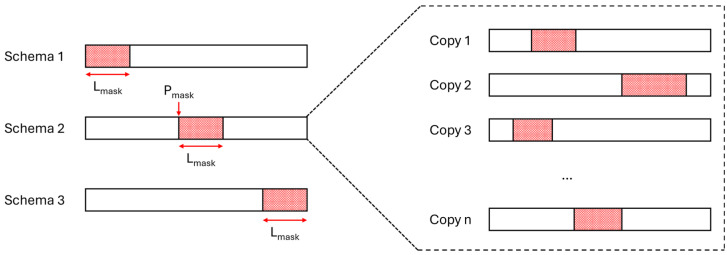
**Left**: Illustration of the three masking schemas for the testing subdatasets. **Right**: Representation of session duplication with different masks sampled from the same distribution.

The lengths of the masked sections (denoted as L_mask_) and the positions of the mask within the session (denoted as P_mask_) for schema 2 were randomly sampled from a uniform distribution. To expand the testing subdatasets, each session was duplicated multiple times, with each duplicate receiving a different mask sampled from the same distribution (see Figure 1).

For each schema, we created two subdatasets: one simulating short GPS outages (10 s < L_mask_ < 20 s) and another simulating medium GPS outages (30 s < L_mask_ < 120 s). The masking process was implemented by setting the following features to null values, preventing the model from accessing this information during localization reconstruction:

- GPS features: P^X^_GPS_, P^Y^_GPS_, P^Z^_GPS_, V^X^_GPS_, V^Y^_GPS_, V^Z^_GPS_, E_GPS;_
- Sensor fusion algorithm output features: P^X^_FUSION_, P^Y^_FUSION_, P^Z^_FUSION_, V^X^_FUSION_, V^Y^_FUSION_, V^Z^_FUSION_, O^A^_FUSION_, O^B^_FUSION_, O^C^_FUSION_, E_FUSION._

### 3.3. Preprocessing

Since the sensor is used across multiple countries, the GPS and sensor fusion algorithm positions (P_GPS_ and P_FUSION_) vary significantly in range. To address this, we converted all positions to relative positions. For the training and validation datasets, as well as the testing subdatasets based on schemas 2 and 3, relative positions were calculated using each session’s first position. For testing subdatasets based on schema 1, relative positions were calculated based on the first position after the masked section.

Next, we applied normalization [17] to scale the features to the range [0, 1]. Additionally, we created a separate velocity vector (a duplicate of V^X^_FUSION_, V^Y^_FUSION_, V^Z^_FUSION_) to serve as the target for training our neural network, with normalization applied in the range [−1, 1]. 

## 4. Approach

### 4.1. Flow

**Figure 2:**
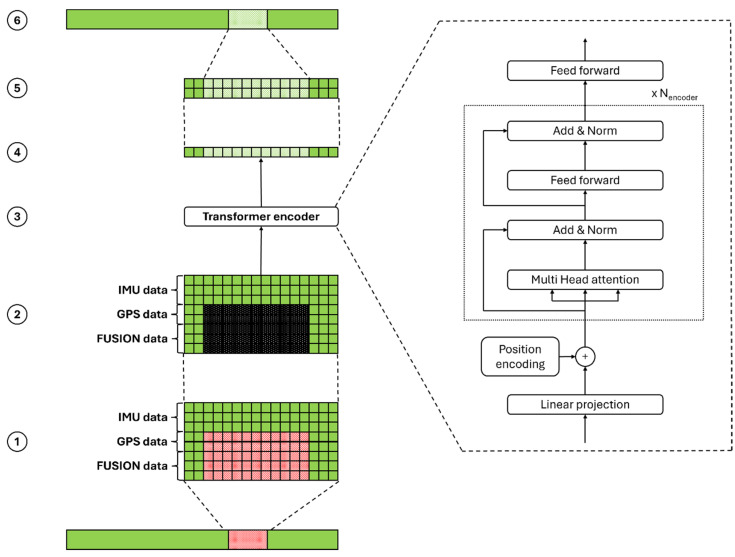
Diagram of the proposed approach. **Left**: (1) A window containing a GPS outage section (highlighted in red) is extracted from the session. (2) GPS and fusion data for timestamps during the outage are masked by replacing values with −1 (highlighted in black). (3) The window is fed into the neural network. (4) The network outputs velocity predictions. (5) Velocities are integrated to compute positions, with an interpolation step applied to ensure smooth transitions for schema 2 (where the GPS outage occurs neither at the beginning nor end of the session). (6) Predicted positions are integrated back into the original session. **Right**: The architecture of the neural network.

To reconstruct the localization of a horse during a GPS outage, we propose a six-step process, illustrated in Figure 2.

**Extracting the GPS outage section**: The GPS outage section is isolated from the session to create a window of size L_seq_, containing all sensor features (listed in Section 3.1. Data) at a frequency of 100 Hz. For short GPS outages (up to 20 s), we define L_seq_ = 2400 (24 s). For medium GPS outages (up to 120 s), L_seq_ = 12,400 (124 s). This ensures that the window includes at least 4 s of data not recorded during a GPS outage. For outages at the beginning or end of a session, the valid GPS data will be positioned at one extremity of the window. For outages occurring mid-session, valid data will be available at both sides, allowing the network access to past and/or future data to inform its predictions.

**Masking GPS outage timestamps**: Timestamps within the GPS outage are selected, and features dependent on the GPS are masked to signify the outage to the neural network. Masking is applied by replacing values with −1.

**Figure 3:**
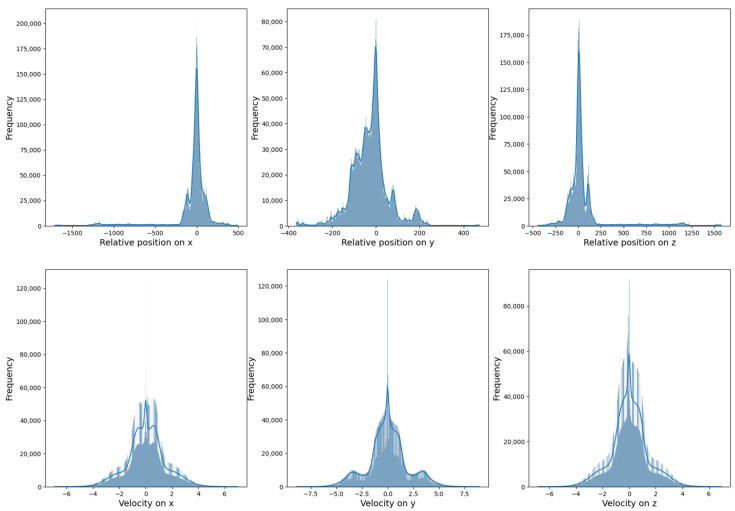
Position in m (**top**) and velocity in m/s (**bottom**) distributions across the full dataset.

**Neural network processing**: The processed window is fed into the neural network, which outputs velocity predictions across three axes. Tests indicated that directly predicting positions resulted in poor performance. As illustrated in Figure 3, velocity distributions are consistently centered around 0 across sessions, while position distributions are highly variable due to differences in session types and distances covered. Therefore, predicting velocities—constrained within a typical range—is more stable than predicting positions, which may vary extensively.

**Integrating velocities**: Positions are derived by integrating the predicted velocities. For outages at the beginning or end of a session, positions are computed unidirectionally. For mid-session outages, a bidirectional integration (forward and backward) is performed, and the results are averaged to distribute position errors more evenly across the GPS outage section.

**Figure 4:**
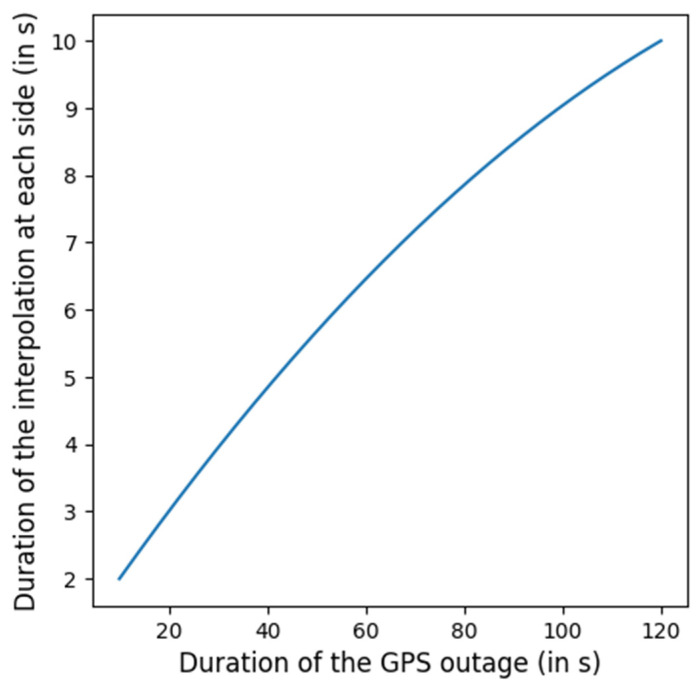
Function defining t_interp_ based on the GPS outage duration.

**Figure 5:**
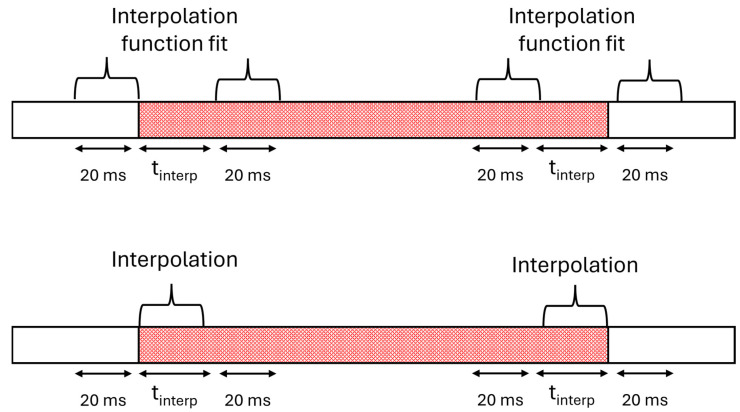
Illustration of the interpolation process following bidirectional integration. **Top**: Quadratic interpolation functions fitted at the boundaries of the GPS outage section. **Bottom**: Interpolations applied to the GPS outage boundaries.

**Applying interpolation (only for GPS outage in the middle of a session)**: Because bidirectional integration can create discontinuities with the rest of the session, an interpolation step is applied at the boundaries of the GPS outage. To achieve smooth junctions, we calculate the duration of data to be interpolated at each extremity of the GPS outage section, denoted as t_interp_ (illustrated in Figure 4). Two quadratic interpolation functions are fitted—one at each boundary. The first function is fitted using data from the 20 ms before the outage and the 20 ms after t_interp_ seconds at the beginning of the outage. The second function uses data from the 20 ms preceding t_interp_ seconds at the end of the outage and the 20 ms following the outage. These functions are then applied to the first and last t_interp_ seconds of the predicted positions. The process is illustrated in Figure 5.

**Merging results**: The final integrated (and interpolated, if applied) positions are merged back into the session.

As we might note, our approach is based on a Transformer network, which poses training challenges due to high computational demands and GPU memory requirements. Given the $O\left(L^{2}\right)$ complexity of Transformers concerning the input sequence length, we also propose a lighter variant of our approach that processes data at a frequency of 10 Hz instead of 100 Hz. In this version, the network outputs the average velocities over 10 timestamps rather than per timestamp.

### 4.2. Network Architecture

Our proposed network architecture is inspired by the BERT model [18]. The network consists of multiple Transformer encoder layers stacked sequentially, followed by a feed-forward layer. To improve precision, the model uses a learnable positional encoding that is updated during training. The architecture is shown in Figure 2. We define d_model_ as the dimension of the embedding, N_head_ as the number of heads in the multi head attentions, d_hidden_ as the size of the inner layer of the feed forwards, and N_encoder_ as the number of encoder layers.

The network takes, as input, a sequence $X\in R^{L_{seq}\times d_{features}}$, where $d_{features}=d_{imu}+d_{gps}+d_{fusion}=9+7+10=26$ is the dimension of the input sequence and L_seq_ is the length of the sequence, as defined in Section 4.1. Flow. Initially, the input sequence is projected into the embedding space $R^{L_{seq}\times d_{model}}$:

$$F_{proj}(X)=XW_{emb}+b_{emb}$$

where $W_{emb}\in R^{d_{features}\times d_{model}}$ and $b_{emb}\in R^{d_{model}}$

Then, the learnable positional encoding is added to the projected input:

$$PE\left(X\right)=X+b_{pe}$$

where $b_{pe}\in R^{{L_{seq}\times d}_{model}}$ is the learnable positional encoding.

After the input projection and positional encoding, the sequence is processed through N_encoder_ encoder layers. Each encoder layer has an identical structure but unique weights and biases. Each layer consists of a residual multi-head attention mechanism followed by a residual feed-forward network.

The residual multi-head attention mechanism, as described by Vaswani et al. in the original Transformer paper [19], is defined as follows:

$$head_{i}\left(X\right)=softmax\left(\frac{XW_{i}^{Q}{\left(XW_{i}^{K}\right)}^{T}}{\sqrt{\frac{d_{model}}{N_{head}}}}\right)XW_{i}^{V}$$

$$MultiHeadAttention\left(X\right)=Concat\left(head_{1}(X),head_{2}(X),\ldots ,{head}_{N_{head}}(X)\right)W^{O}$$

$$X^{’}=LayerNorm(X+MultiHeadAttention\left(X\right))$$

where $W_{i}^{Q}\in R^{d_{model}\times \frac{d_{model}}{N_{head}}}$, $W_{i}^{K}\in R^{d_{model}\times \frac{d_{model}}{N_{head}}}$, $W_{i}^{V}\in R^{d_{model}\times \frac{d_{model}}{N_{head}}}$, and $W^{O}\in R^{{d_{model}\times d}_{model}}$.

The residual feed-forward network, as described by Vaswani et al. in [19], is given by the following:

$$FF\left(X\right)=ReLU\left(XW_{1}+b_{1}\right)W_{2}+b_{2}$$

$$X^{’}=LayerNorm(X+FF\left(X\right))$$

where $W_{1}\in R^{d_{model}\times d_{hidden}}$, $b_{1}\in R^{d_{hidden}}$, $W_{2}\in R^{d_{hidden}\times d_{model}}$, and $b_{2}\in R^{d_{model}}$.

After processing through the N_encoder_ encoder layers, the sequence passes through a final feed-forward network that projects it into $R^{L_{seq}\times 3}$, representing the velocities along the x, y, and z axes for the entire sequence:

$$FF_{final}\left(X\right)=ReLU\left(XW_{1}^{’}+b_{1}^{’}\right)W_{2}^{’}+b_{2}^{’}$$

$$Y=LayerNorm(FF\left(X\right))$$

where $W_{1}^{’}\in R^{d_{model}\times d_{hidden}}$, $b_{1}^{’}\in R^{d_{hidden}}$, $W_{2}^{’}\in R^{d_{hidden}\times 3}$, and $b_{2}^{’}\in R^{3}$.

### 4.3. Network Training

The network was trained for up to 500 epochs, with early stopping applied if no improvement was observed for 20 consecutive epochs [20].

**Figure 6:**
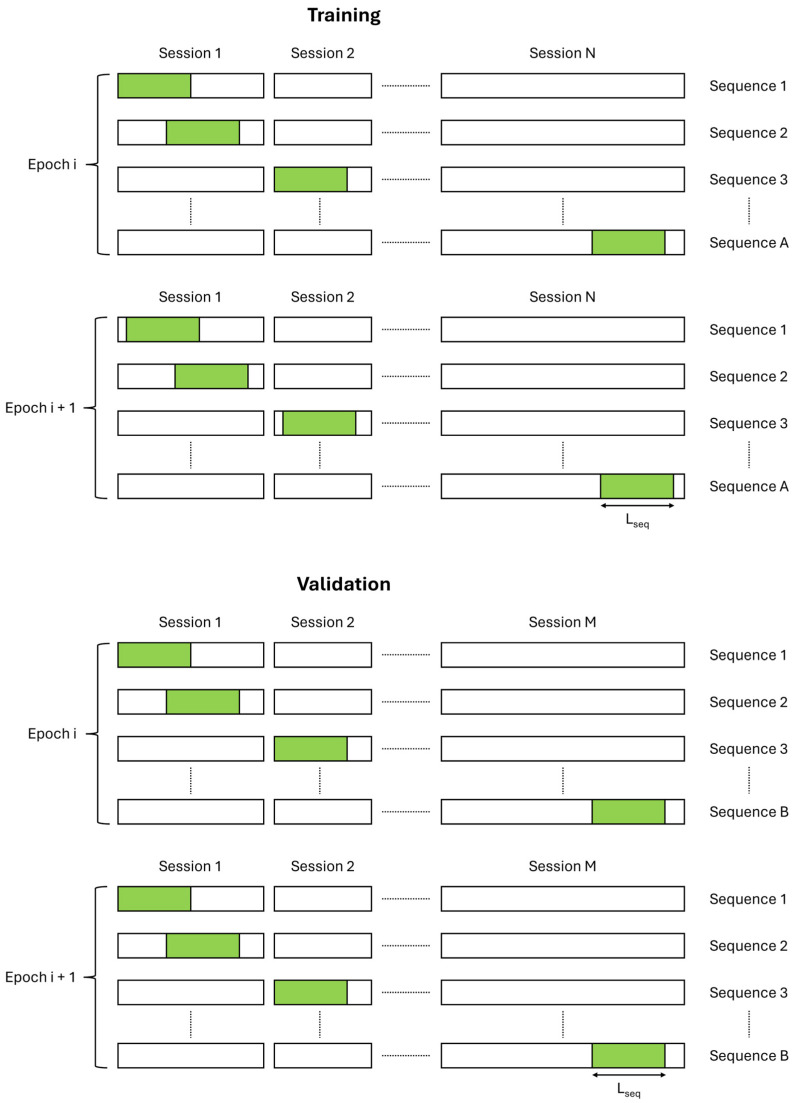
Sequence extraction mechanism for training and validation.

To train and validate the network, sequences of length L_seq_ were extracted from the training and validation sessions. For small GPS outage sections (10 to 20 s), L_seq_ was set to 2400 (or 240 for the lighter network version). For medium GPS outage sections (30 s to 2 min), L_seq_ was set to 12,400 (or 1240 for the lighter version). To extract sequences, we sampled 1/5000th of the timestamps in training sessions as sequence starting points, while 1/1000th of timestamps were sampled in validation sessions. During training, the sampled timestamps were rotated through the dataset every 20 epochs, while validation timestamps remained fixed. This process is illustrated in Figure 6.

Each sequence was assigned a mask of size L_mask_, where L_mask_ was sampled from a uniform distribution. For small GPS outage training, $L_{mask}\;\sim \;U(1000,\;2000)$ (for the lighter version: $L_{mask}\;\sim \;U(100,\;200)$), and for medium GPS outage training, $L_{mask}\;\sim \;U(3000,\;12000)$ (for the lighter version: $L_{mask}\;\sim \;U(300,\;1200)$). We explored several masking strategies (where P_mask_ is the first index of the mask on the sequence):

- **Strategy 1**: Mask applied at the start of the sequence, $P_{mask}=0$;
- **Strategy 2**: Mask applied in the center of the sequence, $P_{mask}=\left(L_{seq}-L_{mask}\right)/\;2$;
- **Strategy 3**: Mask applied at the end of the sequence, $P_{mask}=L_{seq}-L_{mask}$;
- **Strategy 4**: Mask applied at a random position, $P_{mask}\;\sim \;U\left(0,\;L_{seq}-L_{mask}\right).$

Strategies 1, 2, and 3 were tailored for schemas 1, 2, and 3, respectively, while strategy 4 was intended for use across all schemas. During training, the mask length (and position for strategy 4) was regenerated at each epoch, while validation masks remained constant. Masking was performed by setting GPS and sensor fusion features to −1:

- GPS features: P^X^_GPS_, P^Y^_GPS_, P^Z^_GPS_, V^X^_GPS_, V^Y^_GPS_, V^Z^_GPS_, E_GPS_.
- Sensor fusion algorithm output features: P^X^_FUSION_, P^Y^_FUSION_, P^Z^_FUSION_, V^X^_FUSION_, V^Y^_FUSION_, V^Z^_FUSION_, O^A^_FUSION_, O^B^_FUSION_, O^C^_FUSION_, E_FUSION_.

The network was trained to reconstruct the velocity using the Adam optimizer with weight decay [21]. To emphasize minimizing large prediction errors, we selected the Mean Square Error (MSE) loss function. Additionally, we hypothesized that encouraging the network to predict velocity in both the masked and unmasked sections could improve the results by promoting continuity. Therefore, we constructed a dual-objective function that considers both masked and unmasked sections:

$$L_{masked}=\frac{1}{L_{mask}}\sum 1_{i\in M}*{\left(y_{i}-y_{i}^{pred}\right)}^{2}$$

$$L_{unmasked}=\frac{1}{{L_{seq}-L}_{mask}}\sum 1_{i\notin M}*{\left(y_{i}-y_{i}^{pred}\right)}^{2}$$

$$L=\lambda *L_{masked}+{\left(1-\lambda \right)*L}_{unmasked}$$

where M is the masked part of the sequence and λ∈[0;1[ is a hyperparameter, and $y_{i}$ and $y_{i}^{pred}$ are, respectively, the ground truth velocity and the predicted velocity of the ith timestamp of the sequence.

### 4.4. Hyperparameter Tuning

Hyperparameter tuning was conducted using a Bayesian search [22]. The hyperparameter space is detailed in Table 1.

### 4.5. Evaluation

To evaluate our approach, we defined three metrics:

- **The Absolute Trajectory Error (ATE)**: Measures the discrepancy between the ground truth and the predicted trajectories. This metric is sensitive to outliers. Therefore, it is common that this metric increases with the prediction duration and length.
- **The Relative Trajectory Error (RTE)**: Quantifies the relative error in the distance between the ground truth and predicted start/end points.
- **The Relative Distance Error (RDE)**: Calculates the relative error between the total predicted and ground truth distances.

$$ATE=\frac{1}{N}\times \sum_{n=1}^{N}\left|P\left(n\right)-\hat{P}\left(n\right)\right|$$

$$RTE=\frac{\left|P\left(N\right)-P\left(0\right)|-|\hat{P}\left(N\right)-\hat{P}\left(0\right)\right|}{\left|P\left(N\right)-P\left(0\right)\right|}$$

$$RDE=1-\frac{\sum_{n=1}^{N}\left|\hat{P}\left(n\right)-\hat{P}\left(n-1\right)\right|}{\sum_{n=1}^{N}\left|P\left(n\right)-P\left(n-1\right)\right|}$$

where

- $P\left(t\right)$ is the ground truth position in 3D at instant t: (x(t);y(t);z(t));
- $\hat{P}\left(t\right)$ is the predicted position in 3D at instant t: $\left(\hat{x}\left(t\right);\hat{y}\left(t\right);\hat{z}\left(t\right)\right)$;
- $\left|P_{1}\left(n\right)-P_{2}\left(n\right)\right|$ is the Euclidean norm between $P_{1}\left(n\right)$ and $P_{2}\left(n\right)$.

## 5. Results

Our approach achieves an ATE of approximately 3 m for small GPS outages across the entire testing dataset. For medium GPS outages, the ATE is around 12 m. This means that, on average, the predicted positions for small GPS outages are within 3 m of the actual position of the horse, and for medium GPS outages, they are within 12 m. In comparison, state-of-the-art methods based on pedestrian datasets, which feature shorter distances, lower velocities (up to 10 times slower), and reduced acceleration, report ATE values mostly between 4 and 9 m for GPS outages of several seconds to minutes [5,9,10,12].

For the RTE, our approach yields values ranging from 0.2 to 0.7. This implies that the ratio of the distance between the predicted end point and the predicted start point relative to the distance between the actual end point and start point falls between 0.2 and 0.7. In contrast, state-of-the-art methods report RTE values ranging from 0.9 to 6 for one-minute GPS outages in pedestrian contexts [5,9,10,12].

In terms of the RDE, our approach also performs well, averaging 0.3 regardless of the GPS outage duration, highlighting stable performance across various outage lengths. A summary of these results is provided in Table 2 and Table 3.

**Figure A1:**
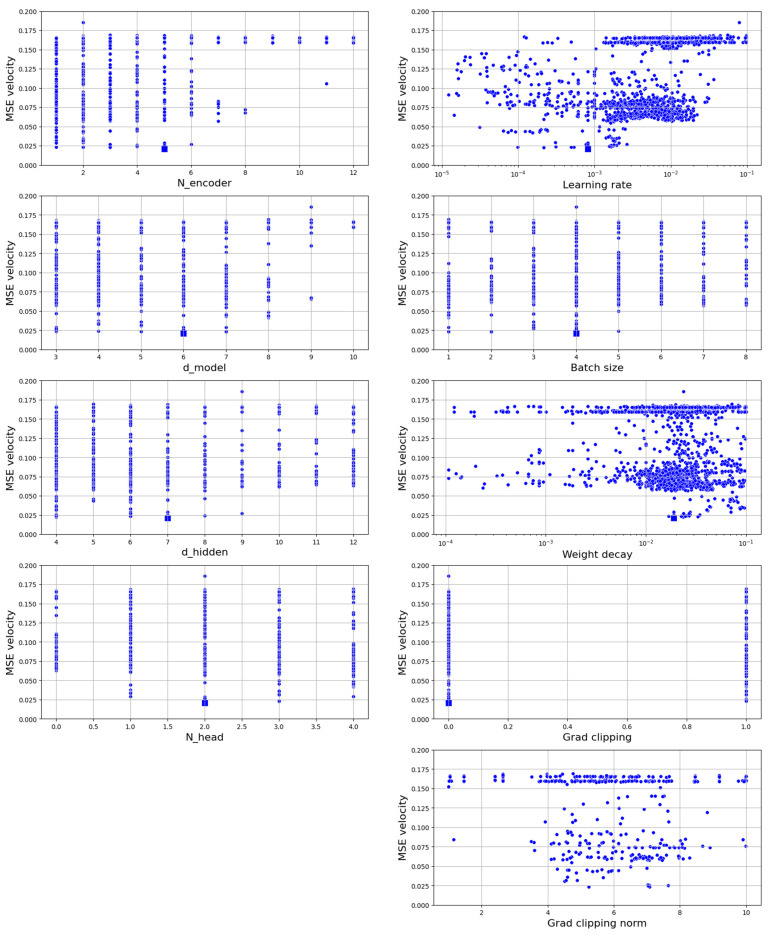
Hyperparameter space versus velocity MSE. Each dot represents a hyperparameter set tested. The square signifies the optimal velocity MSE attained during hyperparameter tuning. The x-scale for the hyperparameters N_encoder_, d_model_, d_hidden_, and N_head_ is in the base-2 logarithm.

Regarding the hyperparameter space, we determined an optimal subspace and the best hyperparameters set, available in Table 4. A more detailed illustration of the results of the hyperparameters search is available in Figure A1.

Another crucial aspect for real-world applications is quantifying the processing time of the deep neural network, which represents the majority of our approach’s computation time, and assessing the memory consumption.

With the best-performing hyperparameter configuration, the network can make an inference on a CPU in 160 ms for small GPS outages and 5.01 s for medium GPS outages. For applications requiring more rapid responses, the lighter network version completes predictions on a CPU in 4.14 ms for small GPS outages and 49 ms for medium GPS outages. On a GPU, prediction times decrease significantly: 3.05 ms (1.76 ms for the lighter version) for small GPS outages and 57.8 ms (1.80 ms for the lighter version) for medium GPS outages. GPU memory consumption is relatively low, with most modern GPUs having ample memory for this task.

Using the best hyperparameter configuration, training on a CPU is impractically slow. However, on a GPU, even a basic model with a few GB of RAM is sufficient. Table 5 provides detailed specifications of the network with the optimal hyperparameters.

For applications requiring hyperparameter searches in the explored space, we recommend a high-performance GPU, as larger networks may demand up to several dozen GB of RAM. Additional information on the processing time and memory usage for various network sizes is available in Appendix C.

**Figure 7:**
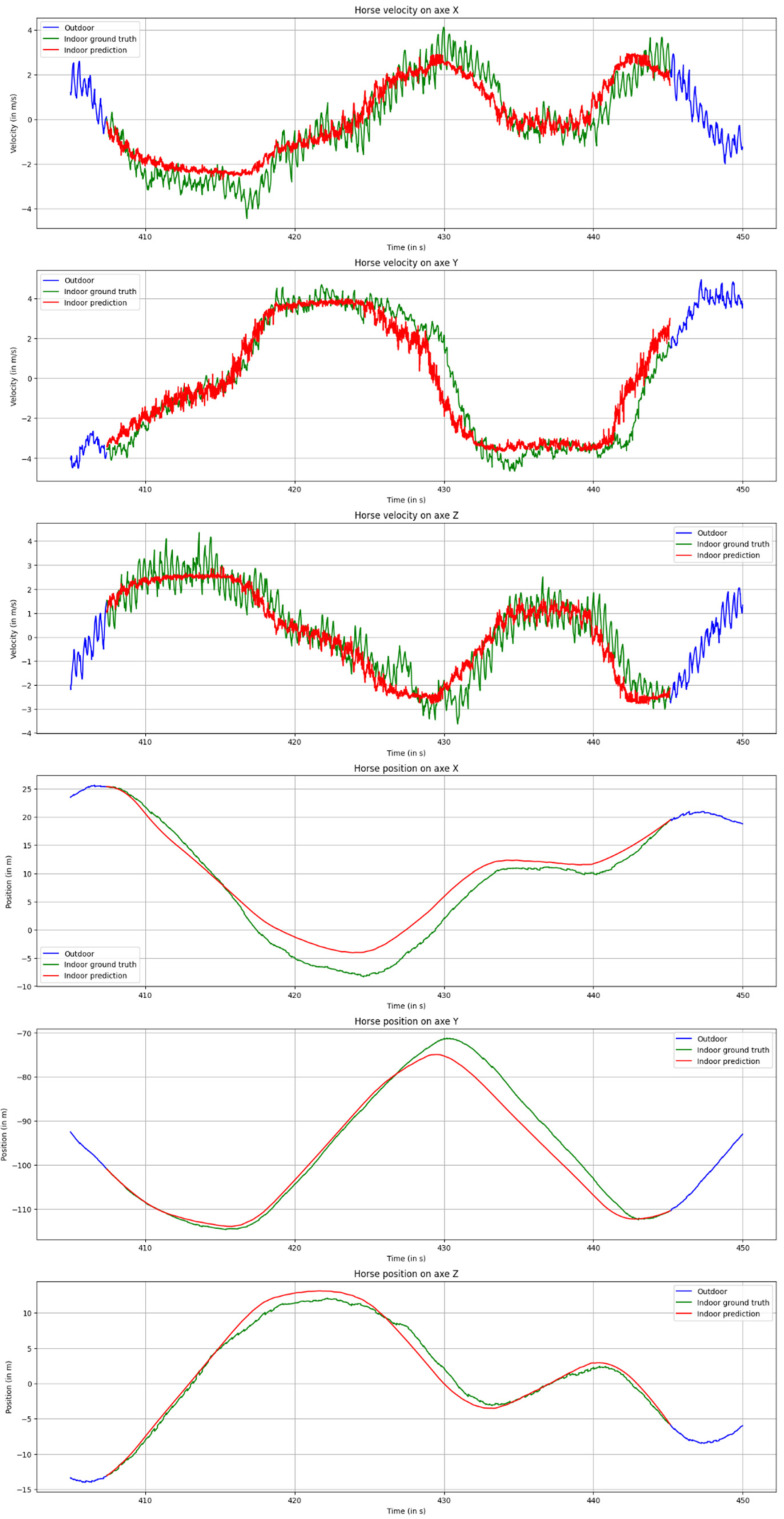
Velocities (first three) and positions (last three) across three axes over time for a 38 s masked section. Green indicates the ground truth (masked before network input to simulate the GPS outage), red indicates network predictions, and blue indicates the unmasked part of the session. Positions are shown post-interpolation.

As shown in Figure 7, our approach accurately predicts the velocity trends and amplitudes, while avoiding adherence to velocity noise, demonstrating the network’s robustness. For position predictions, there is a close alignment with the ground truth signal. Although minor deviations exist at certain points, they appear to be well-balanced by the positive and negative errors within the velocity signals, with occasional accumulations being compensated over time.

**Figure 8:**
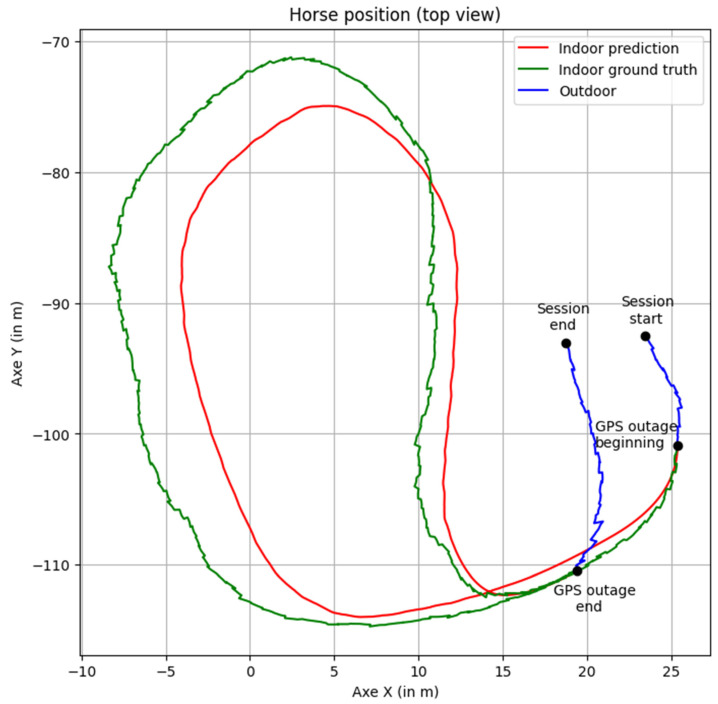
Position on the x-axis versus the y-axis (top view) for a 38 s masked section. Green indicates the ground truth (masked before network input to simulate the GPS outage), red indicates network predictions, and blue indicates the unmasked part of the session. Positions are shown post-interpolation.

From a top-view perspective (illustrated in Figure 8, as seen by riders in the sensor application), the predicted positions align well, and the interpolation step following position integration creates a seamless transition between the GPS outage section and the rest of the session.

## 6. Discussion

**Figure 9:**
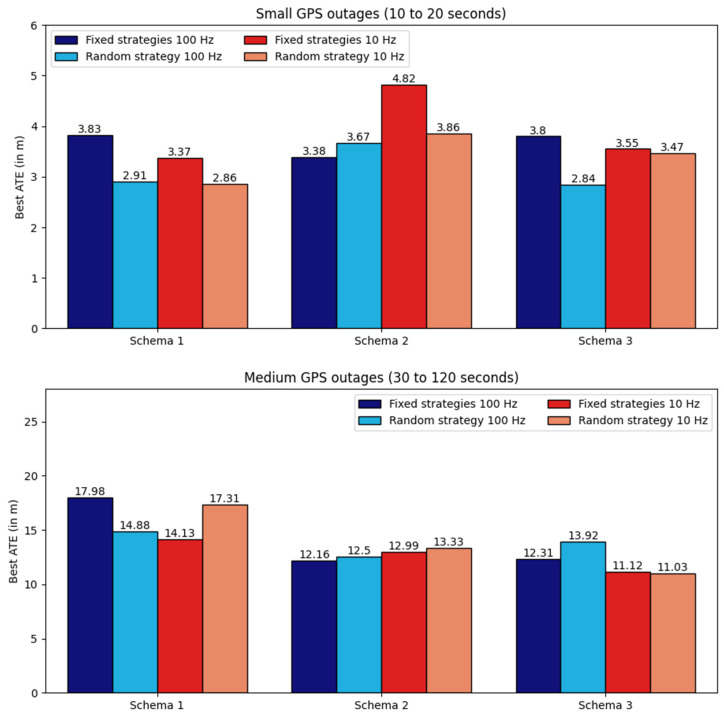
Comparison of the best ATE achieved by the network across three schemas, categorized by strategies and input frequencies. Fixed strategies correspond to strategy 1 for schema 1, strategy 2 for schema 2, and strategy 3 for schema 3. The random strategy represents strategy 4 for all schemas.

In this research, we compared fixed masking strategies (strategies 1, 2, and 3) with a random masking strategy (strategy 4) to train the network. Each fixed strategy is designed for a specific schema, while the random strategy is applicable across all three schemas. On average, both fixed and random strategies produced nearly identical results in most cases. Depending on the schema, either a fixed or random strategy yielded the best results (see Figure 9). Thus, while training all four strategies achieves highly accurate results, training only the random strategy can achieve good results with a quarter of the computational cost.

Regarding the input frequency (100 Hz for the base version, 10 Hz for the lighter version), we observed similar results for both, with better outcomes in some cases at 10 Hz (see Figure 9). This may seem surprising at first, but is logical: a lower frequency reduces precision in velocity predictions (predicting the mean over 100 ms rather than every 10 ms). However, velocity errors are well-distributed (balanced between positive and negative errors) over the 100 ms period, so after integration, they cancel out, preventing drift. The only noticeable errors are short-lived (within 100 ms) and correlate with the duration before positive and negative velocity errors balance out. Additionally, the lighter network’s reduced input sequence length allows for faster training, freeing up resources to explore more hyperparameter sets and increasing the chance of finding an optimal configuration. Moreover, even with a GPU with 25 GB of RAM, some configurations remain untested on the base version due to excessive memory requirements, especially for sequences up to 2 min in length.

**Figure 10:**
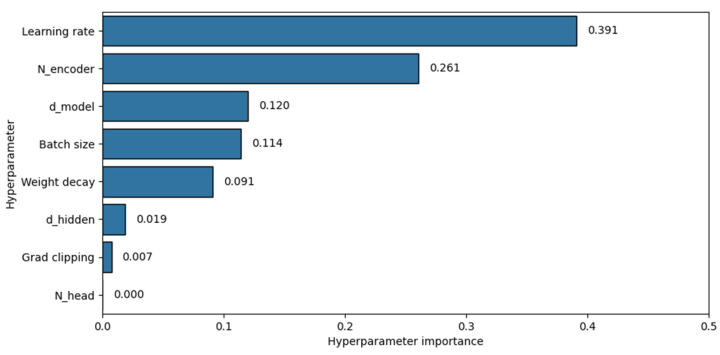
Hyperparameter importance.

We computed hyperparameter importance (shown in Figure 10). For model architecture, the number of encoder layers N_encoder_ and the embedding dimension d_model_ were the most impactful hyperparameters. In the training configuration, the learning rate was the most influential, followed by the batch size and weight decay.

Despite these promising results, several areas of our approach warrant further exploration.

Validation in indoor environments: our approach, designed to predict velocity during GPS outages, was trained on sequences extracted from outdoor sessions. However, horses can exhibit different behaviors (in stride, jump, velocity, and acceleration) between indoor and outdoor environments. Thus, results based on outdoor data do not guarantee accuracy indoors. To validate our approach for indoor use, data would need to be collected in indoor settings, with performance evaluated on masked sequences from those sessions. If indoor performance proves less accurate, retraining the network with both outdoor and indoor data may be necessary. However, because the accurate ground truth cannot be obtained in indoor settings with our sensor due to GPS outages, recording such sessions would require a more sophisticated setup not reliant on GPS and IMU. For example, a combination of UWB and LiDAR could provide the reliable ground truth in indoor environments. We did not conduct this experiment due to the high cost of such setups, which can reach hundreds of thousands of euros.

Computational requirements for longer GPS outages: the computational resources required for training and predicting velocity during GPS outages increase exponentially with the input sequence length. We introduced a lighter version of our approach, which achieved similar accuracy by reducing the input frequency by a factor of ten. However, even with this adjustment, handling large GPS outages (e.g., 5 to 10 min) is challenging and demands a substantial computational infrastructure. Preliminary attempts to reduce the input frequency further (by a factor of hundred) compromised the accuracy. For a longer GPS outage duration, future work could explore advanced attention mechanisms that replicate the performance of standard Transformer attention while reducing computational costs.

Validation across different devices and use cases: we validated our approach on a dataset of horse positions recorded using the Alogo Move Pro device. Future work could expand validation to different devices and various types of motion (e.g., pedestrians, cyclists, racing dogs, drones) to assess the generalizability of the approach.

As the final point of the discussion, we emphasize the significance of the ground truth. Our approach leverages the ground truth velocity as the neural network’s target during training and ground truth position for evaluating localization reconstruction. Two potential sources can serve as the ground truth: (1) the sensor output in contexts where the GPS signal is reliable (the source we used in our study) and (2) a more accurate positioning setup, such as a UWB and LiDAR system. In both cases, the approach learns to reconstruct the ground truth signal in the same manner. The final error in our approach is the sum of two components:

- The error between the reconstructed signal and the ground truth.
- The error between the ground truth and the actual reality.

Both ground truth sources yield similar results for the first error component (reconstruction error relative to the ground truth). However, the second component (ground truth error relative to reality) is likely larger for the sensor-derived ground truth than for the ground truth obtained from a more accurate positioning setup.

Since the conclusions of our study remain valid regardless of the ground truth source, we opted to use the sensor output as the ground truth for budgetary reasons. For industrial applications, however, using a more accurate positioning system may be worth considering. While such setups are more expensive, they could reduce the second error component and yield more precise reconstruction results.

## 7. Conclusions

This paper presents a novel Transformer-based approach to reconstruct indoor localization during postprocessing. Our method leverages a neural network to predict velocity, from which the position is then obtained through bidirectional integration (or unidirectional when bidirectional is not feasible) to better distribute position errors. A final interpolation step ensures smooth transitions between known and predicted positions.

Experiments demonstrate that our approach achieves an ATE of approximately 3 m for short GPS outages (10 to 20 s) and around 12 m for medium GPS outages (30 s to 2 min), which is highly encouraging compared to state-of-the-art results primarily focused on pedestrians rather than horses.

Despite the neural network’s computational complexity, predictions can be made on both a CPU and GPU without specialized requirements. For applications needing rapid predictions, we also propose a lighter version that achieves an accuracy close to the base model while reducing the required sequence length by a factor of ten for the same GPS outage duration. However, training on a CPU remains impractical. Moreover, for hyperparameter exploration, we recommend a high-performance GPU with substantial RAM capacity.

While this research introduces a novel and effective solution that addresses a gap in the existing literature, several areas remain for future exploration: evaluating the model on indoor data acquired with a UWB and LiDAR setup, reducing the model’s computational complexity to handle longer GPS outages, and extending validation to other types of motion beyond horses.

## Figures and Tables

**Table 1:** Hyperparameter space for the Bayesian search.

Hyperparameter	Space
Batch size	[2, 4, 8, 16, 32, 64, 128, 256]
Learning rate	[1 × 10^−5^; 1 × 10^−1^]
Weight decay	[1 × 10^−4^; 1 × 10^−1^]
Gradient clipping	[True, False]
Gradient clipping max norm	[1; 10]
λ	[0.5, 0.75, 0.9, 0.999]
d_model_	[8, 16, 32, 64, 128, 256, 512, 1024]
N_head_	[1, 2, 4, 8, 16]
d_hidden_	[16, 32, 64, 128, 256, 512, 1024, 2048, 4096]
N_encoder_	[1; 12]

**Table 2:** ATE, RTE, and RDE for localization reconstruction of small GPS outages (10 to 20 s). Results for schema 2 are presented before interpolation. The best results per schema and metric are highlighted in bold.

Input Frequence	Schema 1			Schema 2			Schema 3		
	ATE ^1^	RTE ^2^	RDE ^2^	ATE ^1^	RTE ^2^	RDE ^2^	ATE ^1^	RTE ^2^	RDE ^2^
	*Strategy 1*			*Strategy 2*			*Strategy 3*		
100 Hz	3.83	0.62	0.39	**3.38**	**0.20**	**0.28**	3.80	0.88	0.32
10 Hz	3.37	0.41	**0.26**	4.82	0.31	0.29	3.55	0.73	0.32
	*Strategy 4*			*Strategy 4*			*Strategy 4*		
100 Hz	2.91	0.34	0.35	3.67	**0.20**	0.31	**2.84**	0.53	**0.30**
10 Hz	**2.86**	**0.24**	0.38	3.86	0.26	0.33	3.47	**0.51**	0.32

^1^ ATE is expressed in meters; ^2^ RTE and RDE are ratios and do not have units.

**Table 3:** ATE, RTE, and RDE for localization reconstruction of medium GPS outages (30 s to 2 min). Results for schema 2 are presented before interpolation. The best results per schema and metric are highlighted in bold.

Input Frequence	Schema 1			Schema 2			Schema 3		
	ATE ^1^	RTE ^2^	RDE ^2^	ATE ^1^	RTE ^2^	RDE ^2^	ATE ^1^	RTE ^2^	RDE ^2^
	*Strategy 1*			*Strategy 2*			*Strategy 3*		
100 Hz	17.98	0.91	0.30	**12.16**	**0.53**	0.31	12.31	0.35	0.25
10 Hz	**14.13**	**0.73**	0.28	12.99	0.64	**0.30**	11.12	**0.23**	**0.24**
	*Strategy 4*			*Strategy 4*			*Strategy 4*		
100 Hz	14.88	1.08	0.30	12.50	0.55	0.35	13.92	0.34	0.30
10 Hz	17.31	1.18	**0.27**	13.33	0.54	**0.30**	**11.03**	0.28	0.28

^1^ ATE is expressed in meters; ^2^ RTE and RDE are ratios and do not have units.

**Table 4:** Optimal hyperparameter subspace and best hyperparameter set.

Hyperparameter	Optimal Subspace	Best Set
Batch size	[2, 4, 8, 16, 32]	16
Learning rate	[1 × 10^−5^; 5 × 10^−3^]	8.5 × 10^−4^
Weight decay	[1 × 10^−2^; 1 × 10^−1^]	1.95 × 10^−2^
Gradient clipping	False	False
Gradient clipping max norm	None	None
λ	[0.5, 0.75]	0.5
d_model_	[8, 16, 32, 64, 128]	32
N_head_	[2, 4, 8, 16]	4
d_hidden_	[16, 32, 64, 128, 256, 512]	64
N_encoder_	[1; 6]	5

**Table 5:** Processing time and memory consumption for inference (forward pass without gradient computation) with a batch size of 1 and for training (forward pass with gradient computation, backward pass and network optimization) with a batch size of 16. The performances have been established using the best hyperparameters found during the hyperparameter tuning phase (d_model_ = 32, d_hidden_ = 64, N_head_ = 4, and N_encoder_ = 5) on a compute node (4 cores of AMD EPYC-7742 2.25 GHz, 40 Go of RAM, and NVIDIA GeForce RTX 3090).

GPS Outage Duration	Input Frequence	Processing Time on CPU	Processing Time on GPU	GPU Memory Consumption
*Inference (Batch Size 1)*				
small	100 Hz	160 ms/1.61 ms ^1^	3.05 ms/2.09 ms ^1^	36 Mo
small	10 Hz	4.44 ms/217 µs ^1^	1.76 ms/2.29 ms ^1^	32 Mo
medium	100 Hz	5.01 s/521 ms ^1^	57.8 ms/244 µs ^1^	54 Mo
medium	10 Hz	49.0 ms/822 µs ^1^	1.80 ms/2.21 ms ^1^	34 Mo
*Training (Batch Size 16)*				
small	100 Hz	8.42 s/28.0 ms ^2^	30.9 ms/1.43 ms ^1^	304 Mo
small	10 Hz	172 ms/5.35 ms ^2^	16.2 ms/1.40 ms ^1^	74 Mo
medium	100 Hz	None ^3^	527 ms/1.81 ms ^1^	1448 Mo
medium	10 Hz	4.15 s/40.0 ms ^2^	19.1 ms/1.69 ms ^1^	184 Mo

^1^ Mean/standard deviation of 20 loops of 100 repetitions each; ^2^ mean/standard deviation of 20 loops of 10 repetitions each; ^3^ total computation time for 200 repetitions greater than 12 h or memory requirements superior to the configuration.

## Data Availability

The raw data supporting the conclusions of this article will be made available by the authors on request.

## Appendix A

**Table A1:** Statistics of the dataset features (statistics for position features are given after the transformation from the absolute to relative position).

	Mean	Std	Min	Q1	Median	Q3	Max
A^X^_IMU_	−2.35 × 10^−1^	4.53 × 10^0^	−1.10 × 10^2^	−1.95 × 10^0^	−7.50 × 10^−2^	1.80 × 10^0^	1.03 × 10^2^
A^Y^_IMU_	1.32 × 10^−1^	3.32 × 10^0^	−8.73 × 10^1^	−1.53 × 10^0^	1.55 × 10^−1^	1.81 × 10^0^	1.60 × 10^2^
A^Z^_IMU_	−9.97 × 10^0^	6.06 × 10^0^	−1.10 × 10^2^	−1.17 × 10^1^	−9.85 × 10^0^	−8.12 × 10^0^	9.98 × 10^1^
R^A^_IMU_	−8.93 × 10^−2^	2.61 × 10^1^	−3.27 × 10^2^	−1.24 × 10^1^	−4.00 × 10^−2^	1.23 × 10^1^	3.25 × 10^2^
R^B^_IMU_	6.47 × 10^−1^	3.92 × 10^1^	−3.27 × 10^2^	−2.54 × 10^1^	−1.71 × 10^0^	2.49 × 10^1^	3.27 × 10^2^
R^C^_IMU_	−7.51 × 10^−1^	2.74 × 10^1^	−3.08 × 10^2^	−1.81 × 10^1^	−3.90 × 10^−1^	1.68 × 10^1^	3.20 × 10^2^
M^X^_IMU_	−4.09 × 10^−1^	7.57 × 10^1^	−2.36 × 10^2^	−4.99 × 10^1^	−2.65 × 10^0^	4.84 × 10^1^	2.04 × 10^2^
M^Y^_IMU_	6.04 × 10^1^	8.75 × 10^1^	−2.00 × 10^2^	−5.74 × 10^0^	6.09 × 10^1^	1.25 × 10^2^	3.02 × 10^2^
M^Z^_IMU_	−1.78 × 10^1^	1.22 × 10^2^	−2.30 × 10^2^	−1.49 × 10^2^	9.66 × 10^0^	8.06 × 10^1^	2.54 × 10^2^
P^X^_GPS_	−5.64 × 10^1^	2.62 × 10^2^	−1.72 × 10^3^	−4.50 × 10^1^	−2.06 × 10^0^	3.23 × 10^1^	4.97 × 10^2^
P^Y^_GPS_	−2.86 × 10^1^	8.49 × 10^1^	−3.64 × 10^2^	−7.95 × 10^1^	−2.66 × 10^1^	7.44 × 10^0^	4.73 × 10^2^
P^Z^_GPS_	5.90 × 10^1^	2.46 × 10^2^	−4.43 × 10^2^	−2.37 × 10^1^	8.74 × 10^0^	4.75 × 10^1^	1.58 × 10^3^
V^X^_GPS_	7.03 × 10^−2^	1.50 × 10^0^	−7.40 × 10^0^	−7.80 × 10^−1^	4.00 × 10^−2^	9.00 × 10^−1^	9.02 × 10^0^
V^Y^_GPS_	3.40 × 10^−3^	2.01 × 10^0^	−8.36 × 10^0^	−1.02 × 10^0^	−1.00 × 10^−2^	1.03 × 10^0^	8.71 × 10^0^
V^Z^_GPS_	4.79 × 10^−2^	1.45 × 10^0^	−7.68 × 10^0^	−7.40 × 10^−1^	2.00 × 10^−2^	8.20 × 10^−1^	7.61 × 10^0^
E_GPS_	1.50 × 10^0^	2.90 × 10^−1^	9.40 × 10^−1^	1.29 × 10^0^	1.47 × 10^0^	1.66 × 10^0^	4.43 × 10^0^
P^X^_FUSION_	−5.66 × 10^1^	2.62 × 10^2^	−1.71 × 10^3^	−4.49 × 10^1^	−2.19 × 10^0^	3.24 × 10^1^	4.93 × 10^2^
P^Y^_FUSION_	−2.85 × 10^1^	8.48 × 10^1^	−3.64 × 10^2^	−7.93 × 10^1^	−2.63 × 10^1^	7.60 × 10^0^	4.72 × 10^2^
P^Z^_FUSION_	5.88 × 10^1^	2.46 × 10^2^	−4.43 × 10^2^	−2.39 × 10^1^	8.77 × 10^0^	4.61 × 10^1^	1.58 × 10^3^
V^X^_FUSION_	5.93 × 10^−2^	1.44 × 10^0^	−6.91 × 10^0^	−7.50 × 10^−1^	3.00 × 10^−2^	8.50 × 10^−1^	6.99 × 10^0^
V^Y^_FUSION_	4.40 × 10^−4^	2.00 × 10^0^	−8.90 × 10^0^	−1.00 × 10^0^	−1.00 × 10^−2^	1.01 × 10^0^	8.95 × 10^0^
V^Z^_FUSION_	3.72 × 10^−2^	1.39 × 10^0^	−6.85 × 10^0^	−6.90 × 10^−1^	1.00 × 10^−2^	7.70 × 10^−1^	6.92 × 10^0^
O^A^_FUSION_	−1.85 × 10^−2^	9.20 × 10^−2^	−6.43 × 10^−1^	−6.60 × 10^−2^	−1.01 × 10^−2^	4.07 × 10^−2^	7.29 × 10^−1^
O^B^_FUSION_	−2.99 × 10^−2^	7.45 × 10^−2^	−7.33 × 10^−1^	−7.98 × 10^−2^	−3.02 × 10^−2^	1.73 × 10^−2^	9.34 × 10^−1^
O^C^_FUSION_	−6.94 × 10^−2^	1.84 × 10^0^	−3.14 × 10^0^	−1.69 × 10^0^	−8.20 × 10^−2^	1.47 × 10^0^	3.14 × 10^0^
E_FUSION_	4.56 × 10^0^	2.16 × 10^0^	1.60 × 10^0^	3.11 × 10^0^	4.10 × 10^0^	5.28 × 10^0^	1.84 × 10^1^

## Appendix C

**Table A2:** Processing time and memory consumption for inference (forward pass without gradient computation) with a batch size of 1 and for training (forward pass with gradient computation, backward pass, and network optimization) with a batch size of 16. The performances have been established using a small network architecture (d_model_ = 16, d_hidden_ = 32, N_head_ = 2, and N_encoder_ = 3) on a compute node (four cores of AMD EPYC-7742 2.25 GHz, 40 Go of RAM, and NVIDIA GeForce RTX 3090).

GPS Outage Duration	Input Frequence	Processing Time on CPU	Processing Time on GPU	GPU Memory Consumption
*Inference (Batch Size 1)*				
small	100 Hz	41.4 ms/448 µs ^1^	1.64 ms/2.29 ms ^1^	34 Mo
small	10 Hz	1.91 ms/1.41 µs ^1^	1.32 ms/2.29 ms ^1^	32 Mo
medium	100 Hz	809 ms/1.92 ms ^1^	20.7 ms/1.12 ms ^1^	38 Mo
medium	10 Hz	9.15 ms/1.12 ms ^1^	1.42 ms/1.52 ms ^1^	34 Mo
*Training (Batch Size 16)*				
small	100 Hz	4.48 s/116 ms ^2^	14.5 ms/1.70 ms ^1^	142 Mo
small	10 Hz	57.7 ms/4.62 ms ^2^	12.6 ms/1.68 ms ^1^	58 Mo
medium	100 Hz	None ^3^	195 ms/27.3 ms ^1^	584 Mo
medium	10 Hz	1.28 s/29.0 ms ^2^	12.6 ms/1.98 ms ^1^	110 Mo

^1^ Mean/standard deviation of 20 loops of 100 repetitions each; ^2^ mean/standard deviation of 20 loops of 10 repetitions each; ^3^ total computation time for 200 repetitions greater than 12 h or memory requirements superior to the configuration.

**Table A3:** Processing time and memory consumption for inference (forward pass without gradient computation) with a batch size of 1 and for training (forward pass with gradient computation, backward pass, and network optimization) with a batch size of 16. The performances have been established using a medium network architecture (d_model_ = 32, d_hidden_ = 64, N_head_ = 4, and N_encoder_ = 6) on a compute node (four cores of AMD EPYC-7742 2.25 GHz, 40 Go of RAM, and NVIDIA GeForce RTX 3090).

GPS Outage Duration	Input Frequence	Processing Time on CPU	Processing Time on GPU	GPU Memory Consumption
*Inference (Batch Size 1)*				
small	100 Hz	185 ms/21.9 ms ^1^	3.31 ms/1.00 ms ^1^	36 Mo
small	10 Hz	4.71 ms/616 µs ^1^	1.65 ms/1.09 ms ^1^	32 Mo
medium	100 Hz	4.61 s/413 ms ^1^	68.0 ms/1.29 ms ^1^	54 Mo
medium	10 Hz	60.0 ms/4.03 ms ^1^	1.69 ms/966 µs ^1^	34 Mo
*Training (Batch Size 16)*				
small	100 Hz	16.7 s/259 ms ^2^	38.0 ms/1.78 ms ^1^	344 Mo
small	10 Hz	139 ms/3.74 ms ^2^	20.2 ms/2.46 ms ^1^	78 Mo
medium	100 Hz	None ^3^	630 ms/1.36 ms ^1^	1660 Mo
medium	10 Hz	2.92 s/22.1 ms ^2^	20.9 ms/1.82 ms ^1^	206 Mo

^1^ Mean/standard deviation of 20 loops of 100 repetitions each; ^2^ mean/standard deviation of 20 loops of 10 repetitions each; ^3^ total computation time for 200 repetitions greater than 12 h or memory requirements superior to the configuration.

**Table A4:** Processing time and memory consumption for inference (forward pass without gradient computation) with a batch size of 1 and for training (forward pass with gradient computation, backward pass, and network optimization) with a batch size of 16. The performances have been established using a large network architecture (d_model_ = 64, d_hidden_ = 128, N_head_ = 8, and N_encoder_ = 9) on a compute node (four cores of AMD EPYC 7742 2.25 GHz, 40 Go of RAM, and NVIDIA GeForce RTX 3090).

GPS Outage Duration	Input Frequence	Processing Time on CPU	Processing Time on GPU	GPU Memory Consumption
*Inference (Batch Size 1)*				
small	100 Hz	622 ms/20.7 ms ^1^	8.84 ms/1.14 ms ^1^	38 Mo
small	10 Hz	11.2 ms/783 µs ^1^	2.64 ms/1.55 ms ^1^	34 Mo
medium	100 Hz	None ^3^	205 ms/4.17 ms ^1^	54 Mo
medium	10 Hz	176 ms/8.00 ms ^1^	3.05 ms/1.51 ms ^1^	36 Mo
*Training (Batch Size 16)*				
small	100 Hz	None ^3^	98.7 ms/1.58 ms ^1^	1002 Mo
small	10 Hz	873 ms/19.5 ms ^2^	26.1 ms/1.40 ms ^1^	142 Mo
medium	100 Hz	None ^3^	1.87 s/3.39 ms ^1^	4430 Mo
medium	10 Hz	19.5 ms/393 ms ^2^	37.3 ms/1.76 ms ^1^	538 Mo

^1^ Mean/standard deviation of 20 loops of 100 repetitions each; ^2^ mean/standard deviation of 20 loops of 10 repetitions each; ^3^ total computation time for 200 repetitions greater than 12 h or memory requirements superior to the configuration.

**Table A5:** Processing time and memory consumption for inference (forward pass without gradient computation) with a batch size of 1 and for training (forward pass with gradient computation, backward pass, and network optimization) with a batch size of 16. The performances have been established using an extra-large network architecture (d_model_ = 128, d_hidden_ = 256, N_head_ = 16, and N_encoder_ = 12) on a compute node (four cores of AMD EPYC 7742 2.25 GHz, 40 Go of RAM, and NVIDIA GeForce RTX 3090).

GPS Outage Duration	Input Frequence	Processing Time on CPU	Processing Time on GPU	GPU Memory Consumption
*Inference (Batch Size 1)*				
small	100 Hz	1.66 s/138 ms ^1^	22.7 ms/1.02 ms ^1^	38 Mo
small	10 Hz	27.8 ms/274 µs ^1^	3.09 ms/957 µs ^1^	40 Mo
medium	100 Hz	None ^3^	539 ms/9.48 ms ^1^	100 Mo
medium	10 Hz	493 ms/1.45 ms ^1^	6.91 ms/1.47 ms ^1^	42 Mo
*Training (Batch Size 16)*				
small	100 Hz	None ^3^	242 ms/958 µs ^1^	2374 Mo
small	10 Hz	1.78 s/8.73 ms ^2^	27.8 ms/1.33 ms ^1^	308 Mo
medium	100 Hz	None ^3^	4.9 s/8.75 ms ^1^	11,032 Mo
medium	10 Hz	None ^3^	81.7 ms/1.69 ms ^1^	1290 Mo

^1^ Mean/standard deviation of 20 loops of 100 repetitions each; ^2^ mean/standard deviation of 20 loops of 10 repetitions each; ^3^ total computation time for 200 repetitions greater than 12 h or memory requirements superior to the configuration.
